# Digital health and Tourette Syndrome: new technological frontiers in diagnosis and management

**DOI:** 10.3389/fpsyt.2026.1765768

**Published:** 2026-03-24

**Authors:** Cristina Di Vincenzo, Maria Pontillo, Alessandro Antonietti, Alice Cancer, Francesco Demaria, Michelangelo Di Luzio, Deny Menghini, Stefano Vicari

**Affiliations:** 1 Department of Psychology, Università Cattolica del Sacro Cuore, Milan, Italy; 2 Child and Adolescent Neuropsychiatry Unit, Bambino Gesù Children’s Hospital, IRCCS, Rome, Italy; 3 Department of Neuroscience, Università Cattolica del Sacro Cuore, Rome, Italy; 4 Department of Life Sciences and Public Health, Università Cattolica del Sacro Cuore, Rome, Italy

**Keywords:** digital clinical assessment, digital health, digital intervention, internet-delivered behavioural therapy, longitudinal remote monitoring, machine learning, tic disorders, Tourette Syndrome

## Abstract

Tourette Syndrome (TS) is a neuropsychiatric disorder characterized by motor and vocal tics that can significantly impair quality of life. Traditionally, diagnosis and treatment rely on clinical assessments as well as pharmacological and behavioural interventions. In recent years, the advent of digital technologies and machine learning methodologies has opened new possibilities to improve diagnosis, monitoring, and personalized care for patients with TS. This mini-review provides an overview of the main developments in the field, including remote monitoring systems with wearable devices, machine learning predictive models for tic pattern identification, integrated management through eHealth platforms, and digital interventions such as evidence-based online therapy. Current limitations, ethical challenges, and future opportunities are discussed, with particular attention to the multidisciplinary integration of neuroscience, psychiatry, psychology, and digital health. This overview aims to offer an up-to-date foundation to foster the development of innovative and patient-centred strategies in TS management.

## 1 Introduction

Tourette Syndrome (TS) is defined by the presence of multiple motor tics and at least one vocal tic persisting for more than one year with onset in childhood. Tics are sudden, rapid, recurrent, non-rhythmic movements or vocalizations that can be simple or complex, which are often preceded by premonitory urges and can be temporarily suppressed. The clinical course is fluctuating, with typical onset at 5–7 years, a peak around 10–12 years, and spontaneous improvement in many cases during adolescence. Prevalence estimates range from 0.3% to 1%, depending on age (1).

TS is a multidimensional neuropsychiatric condition in which motor/vocal tics frequently co-occur with Attention-Deficit/Hyperactivity Disorder (ADHD), Obsessive-Compulsive Disorder (OCD), anxiety and mood disorders, and Autism Spectrum Disorder (ASD). TS core symptoms and associated comorbidities, together with stigma and limited awareness, substantially affect global functioning and quality of life, making a tic-only diagnostic lens insufficient to capture the disorder’s clinical complexity (1, 2). Longitudinal data suggest a favourable prognosis for most patients. However higher childhood tic severity, neuroanatomical differences in the caudate, and fine motor control deficits predict poorer outcomes. TS itself is associated with adverse clinical trajectories, and comorbid psychopathology (particularly ADHD and OCD) can further worsen these outcomes, underscoring the need for ongoing, individualized follow-up (2, 3).

Standardized assessment relies on clinician-rated and observational tools such as the Yale Global Tic Severity Scale (YGTSS) (4). However, accuracy is limited by intra-individual variability and the waxing and waning of tic severity over time (with natural fluctuations between exacerbations and partial remissions), by context-dependent suppression in the clinic (where patients often inhibit tics when being observed), and by recall bias because ratings frequently depend on brief visits and retrospective parent- or self-reports that systematically misrepresent usual tic frequency and intensity (1). These limitations have driven interest in digital approaches that can deliver objective, ecologically valid, longitudinal measurement. For example, wearable sensor pipelines (EMG/accelerometry) can distinguish tics from voluntary movements with high accuracy (5), while video-based deep learning models achieve automated tic detection and characterization in naturalistic settings, supporting remote monitoring, quantitative severity curves, and early identification (6, 7).

Alongside sharpening assessment and monitoring, technology is increasingly shaping treatment. One example is therapist-supported, internet-delivered exposure and response prevention (ERP), a behavioural intervention in which young people practice repeated tic suppression (response prevention) while premonitory urges are gradually elicited to make suppression more challenging (exposure). Delivered online through 10 structured self-help chapters with brief asynchronous therapist support, ERP has shown good efficacy and acceptability in randomized trials, with signals of cost-effectiveness versus structured education and benefits sustained at follow-up (8–10). A focused review concludes that digital/remote tic interventions can narrow the access gap to evidence-based behavioural therapy (11). Reflecting on these results, the National Institute for Health and Care Excellence (NICE) Early Value Assessment (12) formally evaluates TS-specific digital technologies, including the online behavioural platform named “Online Remote Behavioural Intervention for Tics” (ORBIT) and the Neupulse wearable neuromodulation device, as options for conditional adoption while further evidence is generated (12).

More broadly, the development of digital tools for tic disorders and TS can be situated within broader trends in digital health observed in other movement and neuropsychiatric disorders. Wearable-based monitoring and digital motor assessment have been extensively developed in Parkinson’s disease to quantify motor symptoms and real-world fluctuations (13, 14), with more recent work further supporting the use of digital biomarkers and real-world digital measures (15, 16). In parallel, scalable internet-delivered behavioural interventions have been widely studied across common mental disorders (17, 18), including OCD (19, 20). These methodological trajectories provide a foundation that is now being adapted to the specific phenomenology of tics, including their intermittency, context dependence, suppressibility, and frequent psychiatric comorbidities.

To provide a coherent perspective on the rapidly evolving field of digital health in TS, this mini-review is structured around a clinical digital health framework aligned with the TS care pathway. Specifically, current digital tools are conceptualized across three interrelated domains: (1) digital assessment, aimed at improving objectivity and reliability in tic evaluation; (2) digital monitoring, designed to capture symptom fluctuations over time in ecologically valid, real-world contexts; and (3) digital intervention, intended to expand access to evidence-based behavioural treatments and support long-term management. The main aim of this mini-review is to clarify how digital tools can support objective assessment, enable longitudinal tracking of symptom fluctuations, and expand access to evidence-based behavioural treatment in ways that remain feasible and safe in routine services. Across domains, we synthesise findings by emphasising clinical utility and the main constraints limiting adoption.

## 2 Methods

This manuscript is a mini-narrative review and does not aim to provide an exhaustive or systematic identification of all digital tools for TS. A targeted (non-systematic) literature search was conducted in PubMed and Google Scholar from January 2021 up to November 2025 to focus on the most recent clinically relevant evidence and to identify peer-reviewed studies on digital technologies for the assessment, monitoring, and treatment of tic disorders and TS. Search terms combined keywords related to TS/tic disorders with terms covering the main digital domains addressed here (wearable/sensor-based monitoring, machine-learning approaches, and internet-delivered behavioural interventions). Studies were screened for relevance based on their clinical applicability and contribution to one of the three focal domains (see **Table 1**). Our goal was to identify clinically informative and representative exemplars rather than to map the entire literature. We therefore prioritized: (1) clinical validation studies linking digital outputs to clinician- or expert-rated tic measures; (2) controlled trials of digital behavioural interventions; and (3) health-system or regulatory evaluations relevant to implementation (including NICE Early Value Assessment). Purely technical studies without clinical validation were not prioritized and conference abstracts and other non–peer-reviewed sources were excluded. Selected contextual sources (e.g., clinical guidelines and peer-reviewed overviews) were used to support background, implementation considerations, and limitations.

**Table 1:** Summary of primary studies on digital technologies for assessment, monitoring, and treatment of TS.

Study	Domain	Technology intervention	Study design	Sample	Outcome measures	Key findings	Key limitations/implementation notes
Cernera et al., 2022 (5)	Assessment/Monitoring	Wearable motion sensors	Proof-of-concept observational study	17 TS (8-60y)	Tic *vs* voluntary movement detection; tic frequency *vs* experts	Wearable sensors distinguished tics from voluntary movements with high accuracy (96.7%) and produced tic frequency estimates comparable to expert ratings	Small sample; largely structured recording conditions; limited to motor tics
Rajinikanth et al., 2023 (21)	Monitoring	Multimodal wearable sensor	Prototype proposal + online survey	70 TS (ns)	Perceived usefulness; system description (no clinical performance metrics)	Proposed a multimodal wearable system for real-time detection of “tic attacks” and caregiver alerts; most surveyed users (76%) considered the device potentially useful	No clinical validation; performance unknown; requires individual calibration and usability optimization
Wu et al., 2021 (6)	Assessment	Video-based deep learning	Algorithm development/evaluation	68 TS (4-13y)	Accuracy/precision/recall; tic frequency/area *vs* clinician ratings; time for coding	Deep-learning video analysis detected common facial tics with high accuracy (~95%) and generated tic-frequency estimates comparable to clinician ratings while reducing coding time	Limited labelled data; restricted tic categories; hospital-based recordings
Wu et al., 2023 (7)	Assessment/Monitoring	Video-based tic scoring	Algorithm development/evaluation	57 TS, 44 HC (4-15y)	Accuracy/precision/recall; ROC; time-resolved tic score outputs	Video-based models detected tic events with 91% accuracy and produced time-resolved tic-score curves enabling automated monitoring	False positives in controls; performance tested mainly on specific datasets
Brügge et al., 2023 (22)	Assessment	Video-based motor tic detection	Algorithm development/evaluation	49 TS (11-38y)	Accuracy, ROC-AUC; performance by tic visibility/intensity	Machine-learning models detected facial tics with 88% accuracy, performing best for clearly visible or moderate-severe tics	Limited sample size; restricted to facial motor tics; dependence on high-quality annotations
Andrén et al., 2021 (23)	Intervention	Online ERP, online psychoeducation	Study protocol, single-blind RCT	220 TS/CTD(9–17y)	YGTSS	Describes a randomized trial designed to evaluate the efficacy, durability, and cost-effectiveness of therapist-guided online ERP	Protocol paper; no clinical outcomes available
Hollis et al., 2021 (24)	Intervention	Online ERP	Multicentre single-blind RCT	224 TS/CTD (9–17y)	YGTSS; CGI-I; safety; therapist time	Therapist-supported online ERP produced greater tic reduction than psychoeducation and required substantially less therapist time	Moderate effect size; cannot isolate ERP effects from digital delivery
Hollis et al., 2023 (10)	Intervention	Online ERP	Long-term follow-up of ORBIT	224 TS/CTD (9–17y)	YGTSS; CGI-I; QoL; cost-effectiveness	Long-term follow-up showed sustained improvements up to 18 months and evidence of cost-effectiveness	Naturalistic follow-up; effect sizes slightly reduced over time
Andrén et al., 2022 (8)	Intervention	Online ERP, online psychoeducation	RCT and economic evaluation	220 TS/CTD (9–17y)	YGTSS; CGI-I; satisfaction; cost-effectiveness	Both interventions improved tic severity, but online ERP produced higher treatment response rates than psychoeducation	Active comparator (psychoeducation) produced substantial improvement; short primary follow-up
Rachamim et al., 2020 (25)	Intervention	Internet-based guided self-help CBIT (parent-guided)	Randomized feasibility trial *vs* delayed-treatment control with follow-up	41 TS (15 delayed-treatment control) (7-18y)	YGTSS; CGI-I; functioning/acceptability; AEs	Parent-guided online CBIT produced higher response rates and greater tic reduction than delayed-treatment control with minimal therapist support	Small sample; delayed-treatment control rather than active comparator

TS, Tourette syndrome; CTD, chronic tic disorder; HC, healthy controls; RCT, randomized controlled trial; ERP, exposure and response prevention; CBIT, Comprehensive Behavioural Intervention for Tics; YGTSS-TTSS, Yale Global Tic Severity Scale–Total Tic Severity Score; CGI-I, Clinical Global Impression–Improvement; QoL, quality of life; ROC-AUC, receiver operating characteristic–area under the curve; AEs, adverse events.

## 3 Digital tools for assessment and monitoring tic disorder and TS

In this section, we synthesize digital approaches designed to overcome key limitations of clinic-based tic evaluation, namely, suppression during observation, recall bias, and the waxing–waning course that is poorly captured by brief visits. We focus on wearable- and video-based tools as two complementary routes to objective, time-resolved measurement in real-world contexts. Across approaches, we emphasize (i) clinical validity, (ii) feasibility and burden, (iii) generalisability, and (iv) implementation considerations.

### 3.1 Wearable and sensor-based digital monitoring tools

Wearable sensing approaches are promising for objective tic quantification because they can capture motor physiology continuously and outside the clinic, thereby reducing the impact of suppression and recall bias. Their clinical value depends on whether sensor-derived outputs track clinician-rated severity and whether workflows remain feasible for families and services.

Cernera et al. (5) showed that combined sEMG and accelerometry can differentiate tics from voluntary movements with very high accuracy and generate tic frequency estimates comparable to expert ratings, supporting the feasibility of objective tic monitoring. However, evidence currently relies on small samples and controlled recording contexts, so broader validation in everyday settings (and across diverse tic phenotypes) remains a key next step before routine clinical adoption.

Rajinikanth et al. (21) proposed a multimodal wearable prototype (TSBand) aimed at real-time identification of “tic attacks” and caregiver alerts, suggesting a potential safety/communication function when attacks impair interaction. At present, the system is not clinically validated and requires individual calibration, so its real-world usefulness will depend on reducing setup burden, demonstrating reliability outside laboratory conditions, and clarifying for whom (e.g., paediatric patients with severe attacks) it provides added value.

Overall, wearable systems offer a direct route to continuous, objective tic quantification and could be particularly useful for longitudinal follow-up and outcome tracking. Current limitations are mainly implementation-related (burden/calibration), and evidence gaps include larger real-world validation, performance during complex daily activities, and generalisability to patients with comorbidities and heterogeneous tic presentations.

What clinicians can do now. Wearable systems may currently be considered adjunctive tools in research settings or in selected clinical follow-up scenarios where objective longitudinal quantification is particularly valuable.

What remains investigational. Routine clinical adoption will require larger real-world validation studies, simplified calibration workflows, and clearer guidance on how sensor-derived outputs should inform treatment decisions.”

### 3.2 Video-based and computer vision approaches for tic assessment

Video-based approaches can provide scalable, low-contact assessment by extracting tic-related patterns directly from recordings, with potential advantages for remote monitoring and standardized quantification. Their clinical utility hinges on robustness to naturalistic variability (lighting, camera angle, everyday movements) and on acceptable privacy-by-design solutions, especially for minors.

Wu et al. (6) demonstrated that deep-learning analysis of video can detect common facial tics with high accuracy and produce tic frequency/affected-area estimates comparable to expert ratings, while markedly reducing clinician time for coding. The main constraint is that performance is demonstrated within specific datasets and movement categories; generalisation to broader tic repertoires and real-world variability requires replication across settings and devices.

Wu et al. (7) extended this line by moving toward models that can work with less precisely annotated real-world videos and generate time-resolved outputs that may support monitoring across everyday contexts. While promising for clinical workflows where families record videos at home, the approach still needs clearer evidence on reliability across heterogeneous recording conditions and on how clinicians should interpret and act on model-generated tic curves in practice.

Brügge et al. (22) compared two machine-learning strategies for facial tic detection and found both approaches can reliably distinguish tic from non-tic segments, particularly for more visible tics, supporting feasibility for objective quantification. Misclassifications (e.g., blinking, very mild tics) highlight a clinically relevant boundary condition: video-based tools may be most informative for moderate-to-severe and clearly observable tics, while subtle phenomena remain harder to capture.

Collectively, video-based models support rapid, potentially scalable tic quantification that could reduce clinician burden and enable remote monitoring. Key implementation issues include privacy, data governance, and robustness to everyday recording variability; clinically, future work should test performance across different tic phenotypes, comorbidity profiles, and levels of tic subtlety and establish how outputs map onto clinically meaningful decisions.

What clinicians can do now. Video-based tools may currently support remote follow-up and structured tic quantification, particularly for moderate-to-severe and clearly observable tics, while also reducing clinician coding time in appropriate contexts.

What remains investigational. Broader clinical adoption will require robust validation across heterogeneous real-world recording conditions, as well as clearer guidance on how model-generated outputs should be interpreted and integrated into formal clinical decision-making.

### 3.3 Cross-domain take-home synthesis

Taken together, wearable and video-based tools address complementary limitations of clinic-based tic assessment by providing more objective and time-resolved measures that can be collected outside the clinic. Wearables may offer stronger physiological specificity but can be limited by setup/calibration burden, whereas video tools may be more scalable and lower-burden but face privacy constraints and challenges with subtle/ambiguous movements. In the near term, these technologies are best framed as augmenting, rather than replacing, clinical judgment, with the most immediate clinical role in longitudinal follow-up, outcome monitoring, and supporting treatment planning when clinic ratings are undermined by suppression and recall bias.

Across approaches, clinical validity is most convincing where outputs show agreement with expert/clinician ratings, but evidence remains concentrated in relatively small samples. Feasibility depends on minimizing setup and calibration demands (wearables) and ensuring acceptable, low-effort recording workflows (video). Generalisability remains a priority, particularly across tic phenotypes, levels of subtlety, and comorbidity profiles. Finally, implementation in paediatric care requires privacy-by-design and clear clinical pathways for interpreting and acting on model-generated outputs.

## 4 Integrated care pathway: from measurement to treatment

Digital assessment and monitoring tools become clinically meaningful when their outputs are linked to actionable decisions within care pathways. In practice, repeated ecological measurement can support (i) baseline characterization and shared case formulation, (ii) follow-up and outcome tracking, and (iii) identification of clinically relevant change (e.g., sustained worsening, functional impairment, or treatment non-response) that may indicate the need to intensify care. Conversely, stable or improving symptom trajectories may support maintenance strategies and reduce unnecessary clinic visits.

At baseline, short home videos analysed with computer-vision models can complement clinic ratings, especially when tic suppression is likely. They can show whether tics are mainly context-specific or more pervasive and help set treatment priorities. Wearable sensors (sEMG/accelerometry) can provide objective, physiologically specific estimates of tic frequency and intensity during everyday routines, beyond what recall alone can capture.

For follow-up, videos offer a low-burden option for periodic reassessment, while wearables can track symptoms continuously over days or weeks, which is useful when fluctuations are rapid or treatment response is unclear. Overall, these tools can support personalised care by guiding timely step-up or step-down decisions (e.g., more frequent contact or treatment adjustment when symptoms worsen and maintenance when trajectories are stable).

## 5 Digital interventions for TS treatment

Digital behavioural therapies currently represent the most evidence-supported category of digital health tools for tic disorders and TS. Most studies focus on internet-delivered Exposure and Response Prevention (ERP) and digital or parent-guided adaptations of Comprehensive Behavioural Intervention for Tics (CBIT), with the shared aim of expanding access to evidence-based behavioural treatment while reducing reliance on specialist availability. Across approaches, clinically relevant dimensions include treatment effectiveness and durability, therapist time and feasibility, acceptability, and the extent to which benefits generalise beyond tic severity to broader functioning and comorbid symptoms.

### 5.1 Internet-delivered ERP

Internet-delivered ERP has been developed within structured platforms (e.g., BIP TIC) and evaluated in large randomized controlled trials against active controls. Across trials, therapist-supported online ERP shows clinically meaningful reductions in tic severity and higher early response rates compared with psychoeducation, with relatively low therapist time.

Andrén et al. (23) published a large single-blind RCT protocol that established a rigorous framework for evaluating therapist-guided online ERP versus therapist-supported psychoeducation, including longer follow-up and planned health-economic analyses. This work is important because it positions digital ERP not only as an efficacy intervention but also as a candidate for real-world implementation and cost-effectiveness evaluation.

Hollis et al. (24) reported a large RCT showing that therapist-supported online ERP leads to greater improvements in tic severity and clinician-rated response than an active psychoeducation control, with minimal therapist time and no major safety concerns. These findings support online ERP as a feasible, scalable option for increasing access to behavioural treatment, particularly where specialist services are limited.

Longer-term follow-up from Hollis et al. (10) indicates that benefits are broadly sustained over 12–18 months, including improvements extending beyond tics (e.g., quality of life and functioning), suggesting clinically durable effects rather than short-lived symptom change. However, as with many behavioural interventions, the relative advantage over active controls may attenuate over time, highlighting the importance of identifying which patients benefit most from ERP specifically and whether stepped-care pathways (e.g., starting digitally, escalating to in-person care if needed) optimise outcomes.

Andrén et al. (8) similarly found that both online ERP and structured psychoeducation can yield durable improvements, with ERP showing higher responder rates at earlier time points and signals of advantage on selected secondary outcomes. Follow-up results indicating convergence between groups over time suggest that digital psychoeducation may have non-trivial therapeutic effects, while ERP may be particularly relevant for accelerating clinical response and improving efficiency within service pathways.

Overall, evidence from adequately powered RCTs supports therapist-supported online ERP as an effective and safe intervention that can be delivered with low therapist time. Clinically, its most immediate role is as an accessible first-line or stepped-care option, especially for adolescents and families facing barriers to specialist behavioural therapy; future work should clarify moderators of response (including comorbidity profiles) and determine how best to integrate digital ERP with in-person care when needed.

### 5.2 Digital and parent-guided CBIT interventions

Digital and parent-guided CBIT adaptations aim to preserve core behavioural components while increasing scalability and reducing therapist burden. Early trial evidence suggests feasibility and large symptom improvements, but the evidence base is smaller than for online ERP and would benefit from replication in larger, multi-site studies with active comparators.

Rachamim et al. (25) evaluated an internet-based, parent-guided CBIT self-help program and found high completion/acceptability alongside substantial reductions in tic severity and impairment compared with waitlist, with minimal therapist input. Improvements appeared to extend to comorbid symptoms and functioning in follow-up analyses, although interpretation is limited by sample size and the absence of an active control condition.

Available data indicate that guided digital CBIT formats may offer a scalable route to evidence-based treatment with very low therapist time, potentially fitting well within stepped-care pathways (e.g., parent-guided digital treatment as an entry-level option). Priorities for the field include confirming effectiveness against active controls, clarifying which families are most likely to engage and benefit, and evaluating implementation outcomes in routine services.

### 5.3 Cross-domain take-home synthesis

Taken together, digital behavioural interventions represent the most evidence-supported category of digital health tools for tic disorders and TS and they offer a clear route for translating digital approaches into measurable clinical benefit. Most of the current evidence concerns internet-delivered ERP, supported by large, adequately powered RCTs. On current evidence, therapist-supported online ERP therefore represents the most robustly validated digital behavioural intervention for TS, whereas digital and parent-guided CBIT adaptations show promising but more preliminary findings.

Across interventions, the key clinical and implementation considerations concern the balance between effectiveness and durability of benefit, feasibility (including therapist time), and acceptability, as well as whether improvements extend beyond tic severity to broader functioning and comorbid symptoms. The key implementation question therefore shifts from whether these interventions can be effective in TS overall to how to match the appropriate format and level of therapist support to different patients within stepped-care pathways. Integrating digital treatment with objective monitoring tools may further support outcome tracking and continuity of care, particularly in settings where in-person specialist provision is limited.

Within stepped-care pathways, digital measurement may help operationalize decisions about treatment intensification. For example, insufficient clinical improvement after an initial course of digital therapy, persistently high tic frequency or functional impairment documented through repeated remote assessments, or worsening symptom trajectories may signal the need to increase therapist support or transition to in-person care. Conversely, stable improvement across digital monitoring points may support continuation at the same level of intervention.

## 6 Regulatory evaluation of digital treatments: NICE early value assessment

From a health-system perspective, the NICE Early Value Assessment (12) reviewed two TS-specific digital therapies (see **Table 2**): ORBIT, an online ERP-based guided self-help program, and Neupulse, a wrist-worn device delivering median nerve stimulation.

**Table 2:** Digital technologies evaluated in the NICE early value assessment (12).

Feature	ORBIT	Neupulse
Type of intervention	Digital behavioural therapy (ERP-based, guided self-help)	Neuromodulation device (median nerve stimulation)
Delivery format	Online modules + therapist support (10–20 min/week)	Wrist-worn wearable + smartphone app
Target age group	9–17 years	From 12 years to adulthood
Mechanism of action	Behavioural therapy: exposure with response prevention, psychoeducation, functional analysis	Electrical stimulation modulating sensorimotor networks linked to tics
Regulatory status	Not a medical device. No CE/UKCA marking required	Medical device: CE/UKCA marking pending (expected 2026)
Evidence base	Supported by multiple RCTs (8–10, 24) showing tic reduction and acceptable scalability	Preliminary evidence only; early-phase trials suggesting short-term tic reductions
NICE recommendation	Conditional adoption allowed while more evidence is generated	Not recommended for NHS use at present
Evidence gaps	Long-term outcomes, cost-effectiveness, quality-of-life data	Long-term efficacy, safety, regulatory approval, independent trials
Primary benefits	Improved access to behavioural therapy; low therapist time; high acceptability	Potential rapid tic reduction; portable and easy to use
Primary limitations	Still required therapist engagement; evidence gaps for long-term effects	Insufficient evidence; not yet approved; unclear long-term impact

NICE, National Institute for Health and Care Excellence; EVA, Early Value Assessment; ERP, Exposure and Response Prevention; RCTs, randomized controlled trials; CE, Conformité Européenne; UKCA, United Kingdom Conformity Assessed; NHS, National Health Service.

ORBIT provides an age-appropriate, therapist-supported behavioural intervention over 10 weeks, including psychoeducation, functional analyses, and ERP-based exercises, with 10–20 minutes of therapist contact weekly. Given supportive evidence from United Kingdom (UK) and Swedish trials, NICE concluded that ORBIT may be used within the National Health System (NHS) alongside standard care on a conditional basis, while the developer generates further evidence on long-term effectiveness, quality of life, adherence, adverse events, and cost effectiveness. NICE highlighted that digital behavioural therapies could substantially alleviate the current unmet need, as fewer than 20% of young people in the UK receive evidence-based behavioural treatment for tics.

Neupulse is a wearable neuromodulation device delivering low-intensity electrical pulses to the median nerve to modulate sensorimotor networks implicated in tic generation. Preliminary evidence suggests short-term tic reduction, but long-term clinical data are lacking and the device is still awaiting Conformité Européenne (CE) and United Kingdom Conformity Assessed (UKCA) conformity certification, expected in 2026. Consequently, NICE (12) concluded that Neupulse cannot currently be recommended for NHS use and requires further regulatory and clinical development.

Overall, NICE recognizes the potential of digital therapies to improve access and support behavioural management of tics but emphasizes that routine adoption requires stronger evidence, particularly for neuromodulation-based interventions.

These regulatory and evidence-appraisal considerations help contextualize the current limitations of TS digital technologies, including ethical and practical challenges.

## 7 Limitations and ethical challenges of digital technologies in tic disorders and TS

Although digital technologies for tic disorders and TS show promising potential, several methodological, practical, and ethical limitations emerge across the available evidence.

First, current sensor-based detection systems still face important technical limitations. Wrist-worn systems that combine accelerometry and EMG may inaccurately classify tics when the person is engaged in intense voluntary movements, such as during physical exercise, showing how difficult it can be for these sensors to reliably separate tic-related activity from normal motor behaviour in everyday life (21). In addition, wearable devices often require individualized calibration and continuously collect sensitive motor and physiological data. This raises significant concerns regarding data security, data storage, and the risk of potential re-identification (21), meaning that even anonymized motor or physiological signals may uniquely identify an individual due to their person-specific patterns. Video-based models, including those developed by Wu et al. (6, 7) and Brügge et al. (22), report high accuracy but acknowledge important limitations: difficulty detecting subtle tics, risk of confusion with physiological actions such as blinking, and dependence on training datasets that are relatively small, homogeneous, and limited to specific facial regions or tic types (22).

Second, the generalizability of current findings is limited by the small samples used in most digital intervention studies. Many trials involved modest sample sizes and are conducted within single countries or specialized centres, which restricts cultural and demographic diversity. In addition, several studies include few adult participants or under-represent individuals with common comorbidities (ADHD, OCD, ASD), lack robust quality-of-life measures, and provide incomplete data on long-term maintenance and costs (12).

Third, inequities in digital access may limit real-world scalability. The NICE Early Value Assessment explicitly notes that low digital literacy, lack of stable internet connection, limited access to suitable devices, and cognitive or sensory disabilities can all reduce families’ ability to engage with digital therapies. These structural barriers risk widening existing inequalities, particularly for socioeconomically disadvantaged families or those with limited technological resource (12).

Fourth, adherence and acceptability appear to depend strongly on human support. The narrative review by Khan et al. (11) concluded that digital and remote behavioural therapies achieve higher engagement and more robust clinical outcomes when they are supported by therapists or parents. In contrast, fully self-directed digital formats are associated with lower adherence and reduced effectiveness, suggesting that human guidance remains a critical component for the success of digital interventions (11).

Fifth, ethical challenges, including privacy, autonomy, and stigma, require careful consideration. Continuous behavioural monitoring through video or wearable sensors may feel intrusive for children and adolescents and could increase their self-consciousness about tics. Importantly, privacy concerns in this context extend beyond technical data security risks (e.g., breaches, storage, re-identification) to include psychosocial dimensions of acceptability, such as perceived intrusiveness, impact on autonomy, and the child’s subjective experience of being continuously monitored. NICE has also highlighted concerns that visible devices such as Neupulse might attract unwanted attention and contribute to social stigmatization (12). Moreover, many digital studies provide limited information on data governance frameworks and consent procedures, despite involving minors and highly sensitive behavioural data. Finally, major evidence gaps remain regarding long-term outcomes, safety, and cost-effectiveness. NICE highlighted insufficient data on the durability of clinical benefits, quality-of-life impacts, adverse events, and real-world cost implications for both ORBIT and Neupulse, and therefore called for further evidence generation before these interventions can be routinely adopted within the NHS (12).

Similarly, although short-term improvements have been demonstrated in ERP and ICBIT trials, data beyond 6–12 months are still limited and the impact of digital interventions on comorbidities, functional outcomes, and relapse trajectories remains poorly characterized.

This mini-review addresses different digital health technologies that involve distinct ethical implications depending on their use (e.g., monitoring/assessment versus intervention). We therefore recommend that these ethical implications be considered in a technology-specific manner, taking into account the context of use.

## 8 Conclusion and future directions

Digital health technologies offer new ways to assess and treat tic disorders and TS by extending measurement and support beyond the clinic. Taken together, current findings suggest that the most immediate contribution of these technologies is not to replace clinical judgement, but to strengthen it by improving the objectivity and continuity of tic monitoring and by expanding access to evidence-based behavioural treatment through structured, scalable formats.

At the same time, the evidence base is not yet mature enough to support routine implementation. Promising results are often derived from small or selected samples, with limited information on durability, performance across comorbidity profiles, and real-world effectiveness. Implementation will also hinge on resolving key ethical and equity issues, including privacy and data governance, the risk of stigma linked to visible monitoring, and unequal access to devices, connectivity, and digital literacy. In line with NICE guidance, robust data on long-term clinical effectiveness, quality-of-life outcomes, adherence, and cost-effectiveness remain prerequisites for widespread adoption. Future research should therefore prioritise (1) larger, multisite studies designed for generalisability; (2) multimodal, person-level modelling of tic dynamics through integrated data streams (e.g., wearables, video, ecological momentary assessment, and patient-reported outcomes); and (3) implementation-focused evaluations of blended care pathways that combine digital tools with clinician oversight. Overall, digital technologies are best positioned as complements within multidisciplinary care, enhancing monitoring and widening access, provided they are supported by rigorous validation, ethical safeguards, and practical strategies that make them usable for families in everyday settings.
